# Leveraging circulating microbiome signatures to predict tumor immune microenvironment and prognosis of patients with non-small cell lung cancer

**DOI:** 10.1186/s12967-023-04582-w

**Published:** 2023-11-10

**Authors:** Xiaohan Zhou, Liting You, Zhaodan Xin, Huiting Su, Juan Zhou, Ying Ma

**Affiliations:** 1 Department of Laboratory Medicine, West China Hospital, Sichuan University, Chengdu, 610041 People’s Republic of China; 2 Laboratory of Aging Research and Cancer Drug Target, State Key Laboratory of Biotherapy, National Clinical Research Center for Geriatrics, West China Hospital, Sichuan University, Chengdu, 610041 People’s Republic of China; 3 Department of Laboratory Medicine, Guang ‘an People’s Hospital, Guang ‘an, 638000 Sichuan People’s Republic of China

**Keywords:** Circulating microbiome, Non-small cell lung cancer, Prognostic biomarkers, Tumor immune microenvironment, Drug sensitivity

## Abstract

**Background:**

Accumulating evidence supports the significant role of human microbiome in development and therapeutic response of tumors. Circulating microbial DNA is non-invasive and could show a general view of the microbiome of host, making it a promising biomarker for cancers. However, whether circulating microbiome is associated with prognosis of non-small cell lung cancer (NSCLC) and its potential mechanisms on tumor immune microenvironment still remains unknown.

**Methods:**

The blood microbiome data and matching tumor RNA-seq data of TCGA NSCLC patients were obtained from Poore’s study and UCSC Xena. Univariate and multivariate Cox regression analysis were used to identify circulating microbiome signatures associated with overall survival (OS) and construct the circulating microbial abundance prognostic scoring (MAPS) model. Nomograms integrating clinical characteristics and circulating MAPS scores were established to predict OS rate of NSCLC patients. Joint analysis of blood microbiome data and matching tumor RNA-seq data was used to deciphered the tumor microenvironment landscape of patients in circulating MAPS-high and MAPS-low groups. Finally, the predictive value of circulating MAPS on the efficacy of immunotherapy and chemotherapy were assessed.

**Results:**

A circulating MAPS prediction model consisting of 14 circulating microbes was constructed and had an independent prognostic value for NSCLC. The integration of circulating MAPS into nomograms may improve the prognosis predictive power. Joint analysis revealed potential interactions between prognostic circulating microbiome and tumor immune microenvironment. Especially, intratumor plasma cells and humoral immune response were enriched in circulating MAPS-low group, while intratumor CD4 + Th2 cells and proliferative related pathways were enriched in MAPS-high group. Finally, drug sensitivity analysis indicated the potential of circulating MAPS as a predictor of chemotherapy efficacy.

**Conclusion:**

A circulating MAPS prediction model was constructed successfully and showed great prognostic value for NSCLC. Our study provides new insights of interactions between microbes, tumors and immunity, and may further contribute to precision medicine for NSCLC.

**Supplementary Information:**

The online version contains supplementary material available at 10.1186/s12967-023-04582-w.

## Introduction

Lung cancer ranks first in mortality and second in morbidity among malignant tumors worldwide [1]. Among them, non-small cell lung cancer (NSCLC) accounts for over 85% in terms of pathological types. Despite novel advancements in therapeutic strategies, the 5 year overall survival (OS) rate for NSCLC patients remains at a low level (approximately 15%) [2]. There is an urgent need to identify novel biomarkers associated with the prognosis and therapeutic efficacy of NSCLC patients and reveal the potential mechanisms.

Microbiota have recently emerged as novel tumorigenesis regulators and biomarkers in multiple types of cancers, including lung cancer [3]. Microbiome dysbiosis contributes to cancer susceptibility and progression through complex mechanisms, including promoting inflammatory, producing toxins, causing DNA damage, activation of cancer-related pathways, etc. [3–7]. Besides, increasing evidence indicated that the enormous microbiome of host contributes to maintaining host-immune balance and shaping tumor microenvironment, thus to cancer progression and treatment response [8, 9]. With the rise of high-throughput sequencing technology, the microbiome has shown broad prospects for cancer diagnosis and treatment. Several studies have demonstrated the globe changes of lung microbiome between healthy participants and lung cancer patients, and between patients with different prognosis [10, 11]. However, specimens from lung, such as lung biopsy tissues and bronchoalveolar lavage fluid, are not always readily available.

After ruling out potential contaminations, researchers have identified circulating microbial DNA (cmDNA) from cancer patients’ peripheral blood [12, 13]. Peripheral blood flows through the whole body and can carry molecules from various parts of the body, including tumor tissues. Therefore, theoretically, cmDNA could show a general view of the microbiome of host. In addition, cmDNA has great advantages for clinical application, including non-invasive operations, making it a promising biomarker for cancer diagnosis and progression [13]. Emerging evidence has identified the characteristic composition patterns of cmDNA in various cancers, including early-onset breast cancer, prostate cancer, colorectal cancer, pancreatic cancer, and hepatocellular carcinoma [12, 14–17]. In addition, Messaritakis et al. demonstrated the clinical value of cmDNA in predicting cancer progression and prognosis in CRC patients. They found that 16S rDNA, the β-galactosidase gene of *Escherichia coli*, glutamine synthase gene of *Bacteroides fragilis*, and 5.8S rRNA of *Candida albicans* in peripheral blood were associated with the metastatic disease and shorter survival rates [18]. However, whether circulating microbiome signatures are associated with prognosis of NSCLC and its potential mechanism on tumor immune microenvironment still remains unclear.

In this study, we obtained the blood microbiome data and the matching tumor RNA-seq data of TCGA NSCLC patients, to dissect their profiling and key roles in NSCLC. We aimed to establish a novel and comprehensive circulating microbial abundance prognostic scoring (MAPS) model to decipher the tumor immune microenvironment and to predict the efficacy of chemotherapy and immunotherapy and the prognosis of NSCLC patients. We believe that novel explorations of circulating microbiome signatures will provide a new idea of interactions between microbes, tumors, and immunity, and provide significant clues for development and intervention of NSCLC patients, which will further promote the development of precision medicine for NSCLC.

## Materials and methods

### Data acquisition

**Fig. 1:**
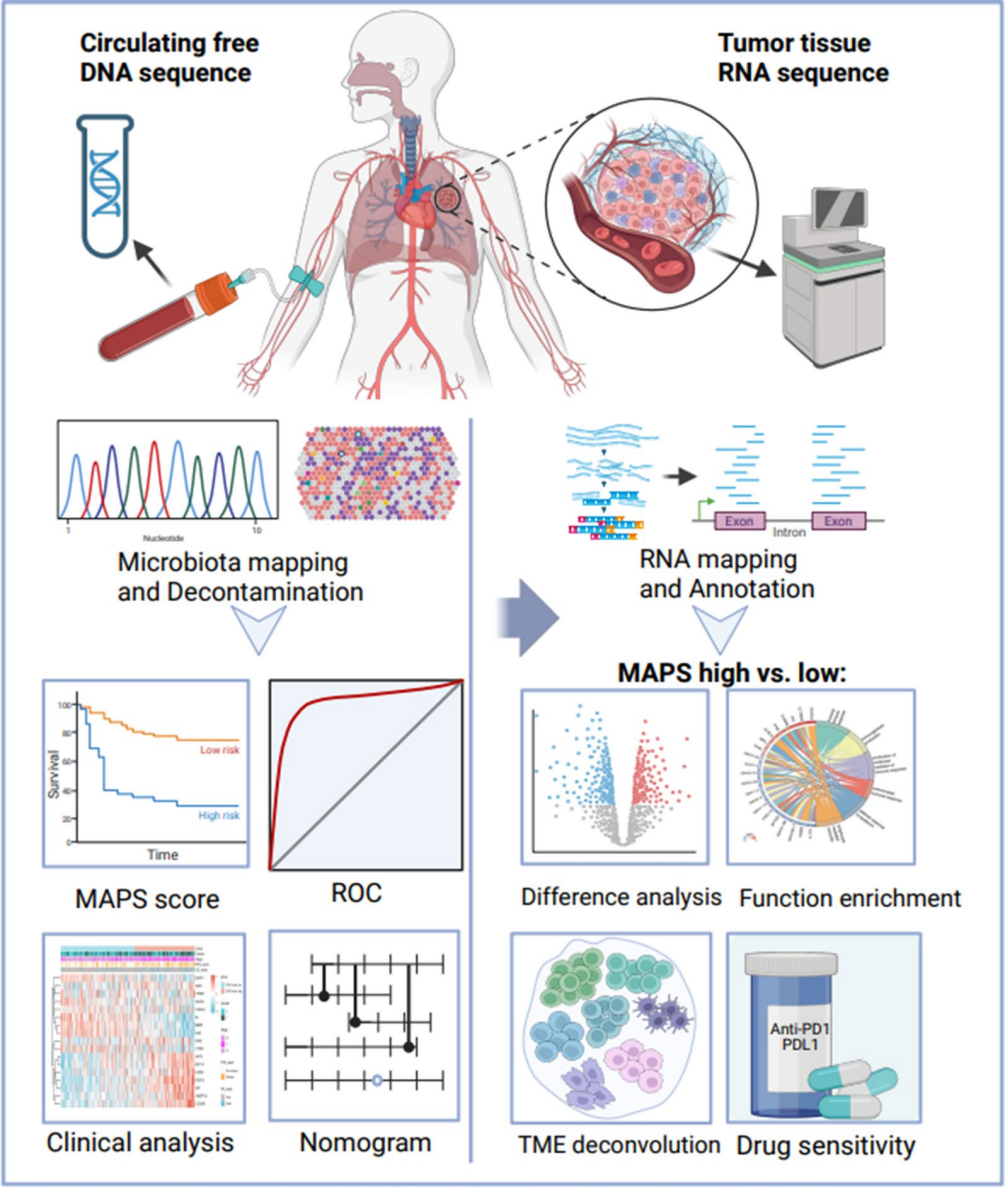
The workflow for comprehensive analysis of circulating microbiome in TCGA NSCLC patients

The workflow of this study is demonstrated in Fig. 1. All NSCLC patients in TCGA with blood microbiome data and survival information of any stages and grades (n = 109) were included in this study. For 109 NSCLC patients in TCGA, the clinical data, survival data, and both blood microbiome data and matching tumor RNA-seq data (gene counts) were downloaded for this study in December 2022 (see Additional file 1). Briefly, the NSCLC blood microbiome data was downloaded from Poore’s study [19], where a total of 1553 genera were detected and quantified after Voom-SNM normalization and removing the potential contaminants. To describe the tumor microenvironment landscape of patients with NSCLC in TCGA, the matching tumor RNA-seq data (gene counts) and clinical data including survival information were obtained from UCSC Xena [20]. Transcripts per million (TPM) data was transformed from gene counts data based on gene effective length.

### Construction of circulating microbial abundance prognostic scoring model

The TCGA NSCLC blood microbiome data of 109 samples was introduced to select the circulating microbial prognostic signatures to construct the good-performance prognostic model by using “survival” and “survminer” package in R. First, use univariate cox regression analysis to identify the candidate circulating microbial signatures that significantly associated with OS of patients (*P* < 0.05). Among them, multivariate cox regression analysis was then performed to select the circulating microbial prognostic signatures that independently impact OS (*P* < 0.05). Finally, the circulating microbial abundance prognostic scoring (MAPS) model of each patient was calculated by the linear combination of the OS-related microbes’ abundance and multivariate cox regression coefficient [21]:

$$\mathrm{MAPS}={\sum }_{i=1}^{14}\left(risk\,coefficient\,of\,microbe\,i\right)*(abundance\,of\,microbe\,i)$$

All patients were divided into circulating MAPS-high and MAPS-low groups according to the optimal cutoff value determined by the “surv_cutpoint” function. Kaplan–Meier curves and ROCs were used to assess the performance of circulating MAPS for predicting OS. Then, we conducted clinical relevance analyses between collected clinical characteristics (age, gender, T stage, N stage, stage and status) and circulating MAPS.

### Nomogram

To assist in predicting the 1-, 3- and 5 year OS rate of NSCLC patients, clinical characteristics (age, subtype, gender and stage) and circulating MAPS scores were integrated to build the prognostic nomogram model by using the “rms” package. The time-dependent ROC and concordance index (C-index) was used to indicate model performance.

### Joint analysis of tumor microenvironment and circulating microbiome

To describe the tumor microenvironment landscape of patients in circulating MAPS-high and MAPS-low groups, matching tumor RNA-seq data of these two subtypes was introduced. First, we identified the overall differentially expressed genes (DEGs) between these two groups by using “DESeq2” package. The KEGG and GO gene list was obtained from MSigDB (https://www.gsea-msigdb.org/gsea/msigdb/). And “clusterProfiler”, “enrichplot”, and “fgsea” packages were used to perform Gene Set Enrichment Analysis (GSEA). Then, according to the ImmPort database (https://www.immport.org/home), immune-related DEGs were annotated and reflected to GO and KEGG items. The univariate cox regression analysis and Kaplan–Meier survival curves were used to evaluate the prognostic value of the immune-related DEGs. And we further deciphered the underlying relevance of the immune-related DEGs and the circulating microbial prognostic signatures. Furthermore, the “xcell” method in “immunedeconv” package was used to assess the immune infiltration in tumor. Finally, we compared the expression levels of immune checkpoint molecules (PD-L1 and CTLA4) between these two groups.

### Drug sensitivity

In this study, we investigated the predictive value of circulating MAPS on the efficacy of immunotherapy and chemotherapy. Tumor Immune Dysfunction and Exclusion (TIDE; http://tide.dfci.harvard.edu/) score was used to predict immunotherapy responses of each patient. The “oncoPredict” package in R was applied to estimate the drug’s half maximal inhibitory concentration (IC50) and predict the chemotherapy drug sensitivity of each patient.

### Statistical analysis

All statistical analyses and graphic visualizations were performed in R (version 4.2.2). Student’s t-test or Wilcoxon rank sum test were used to compare continuous variables between groups. *P* < 0.05 was considered statistically significant (two-tailed).

## Results

### Circulating microbiome signatures were associated with NSCLC prognosis.

**Fig. 2:**
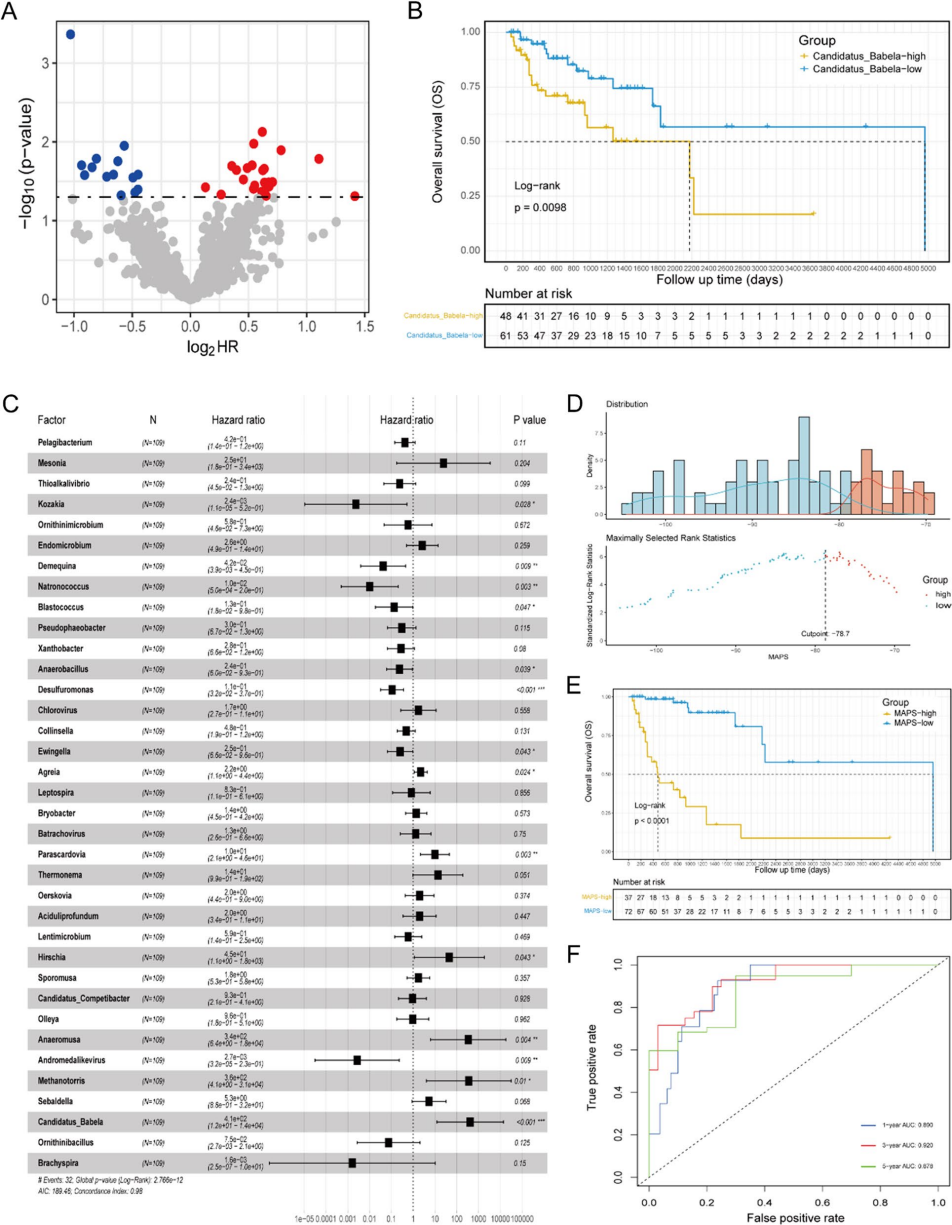
Construction of the circulating MAPS model for prognosis of NSCLC patients. **A** Volcano plot of the candidate circulating microbial signatures that significantly associated with OS by a univariate cox regression. **B** Kaplan–Meier OS curve of the representative microbe (*Candidatus_Babela*). **C** Forest plot of the circulating microbial prognostic signatures that independently impact OS by a multivariate cox regression. **D** Optimal cutoff value of circulating MAPS determined by the “surv_cutpoint” function. **E** Kaplan–Meier OS curve of circulating MAPS. The *P* value was calculated by log-rank test between circulating MAPS-high and MAPS-low groups. **F** ROC illustrated the performance of circulating MAPS for predicting the 1-, 3- and 5 year OS rate

In order to elucidate the prognosis prediction value of circulating microbiome signatures in NSCLC patients, a TCGA NSCLC dataset with blood microbiome data was introduced. For a total of 1553 genera detected from 109 samples (Additional file 1), the normalized microbial abundance data by Voom-SNM method ranges from − 10.83 to 24.16, with an average of 1.06. Firstly, we identified 36 genera that significantly associated with OS of NSCLC patients according to the univariate cox regression analysis. (Fig. 2A, B). Among them, 22 microbes were considered as risk factors (hazard ratio (HR) > 1; *P* < 0.05) while 14 microbes were considered as favorable factors (HR < 1; *P* < 0.05). We further identified 14 OS-related genera that independently impact OS using multivariate cox regression analysis (Fig. 2C). *Candidatus_Babela*, *Methanotorris*, *Anaeromusa*, *Hirschia, Parascardovia*, *Agreia* were defined as prognosis risk factors, while *Kozakia*, *Andromedalikevirus*, *Natronococcus*, *Demequina*, *Desulfuromonas*, *Blastococcus*, *Anaerobacillus*, *Ewingella* were defined as prognosis favorable factors.

Furthermore, a circulating microbial abundance prognostic scoring (MAPS) model was constructed to assess the patients’ death risk by the linear combination of the above 14 microbes’ abundance and multivariate cox regression coefficient (see Additional file 2). Based on the optimal cutoff value of circulating MAPS determined by the “surv_cutpoint” function (Fig. 2D), patients were divided into circulating MAPS-high group and MAPS-low group. The Kaplan–Meier curve and log-rank test indicated that NSCLC patients with high circulating MAPS had a shorter OS than those with low circulating MAPS (Fig. 2E). The AUCs of circulating MAPS for predicting the 1-, 3- and 5 year OS rate were 0.890, 0.920, and 0.878, respectively (Fig. 2F).

### Circulating MAPS was an independent prognostic indicator of NSCLC patients

**Fig. 3:**
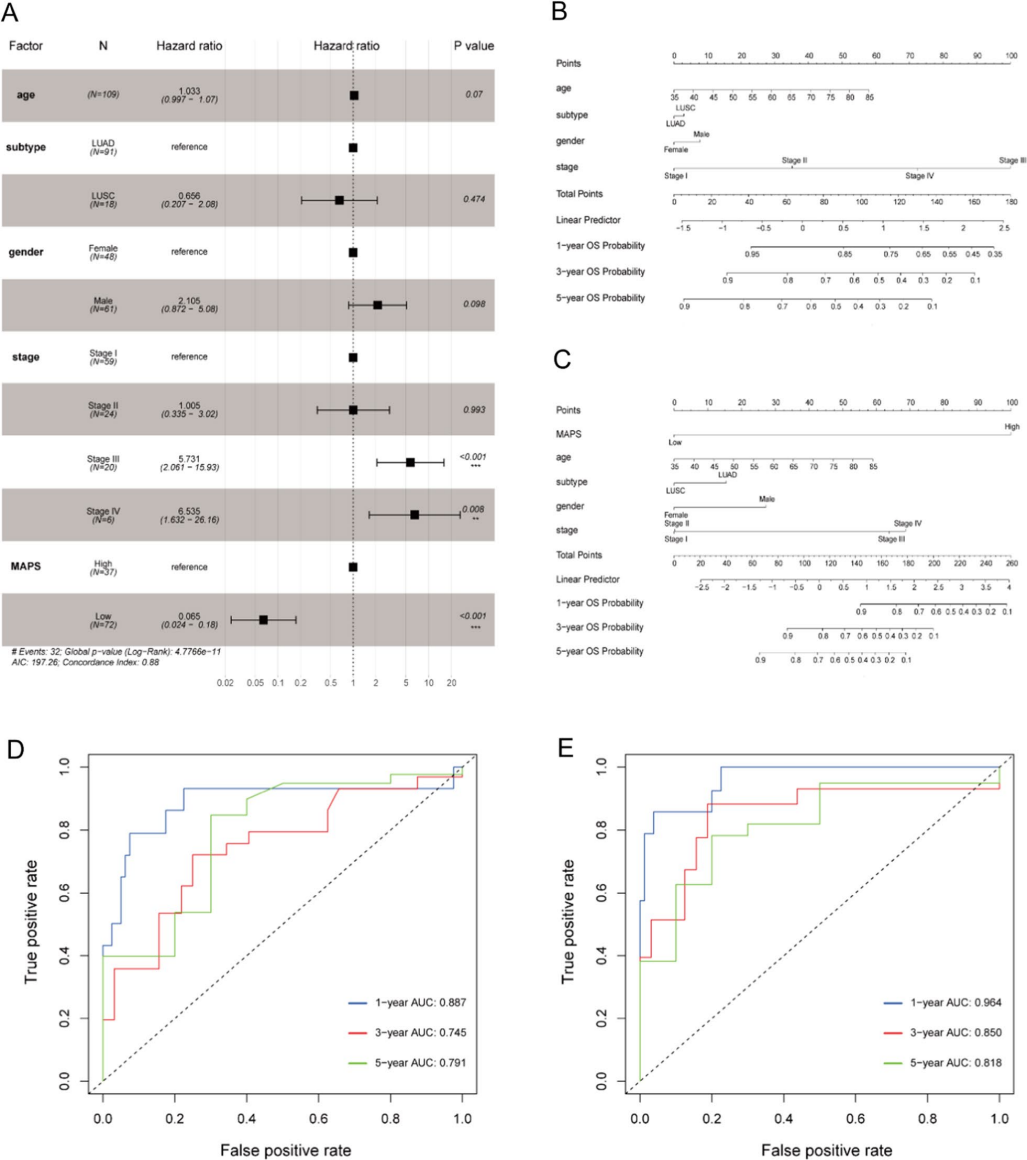
Development of the nomogram survival model based on circulating MAPS. **A** Forest plot illustrated the independent prognostic impact of circulating MAPS by adjusting for clinical characteristics (including age, subtype, gender and stage). **B**, **C** Nomogram models based on clinical factors without **B** or with **C** circulating MAPS to predict the 1-, 3- and 5 year OS probability of NSCLC patients. **D, E** Time-dependent ROCs illustrated the performance of the nomogram models without **D** or with **E** circulating MAPS

Multivariate cox regression analysis based on circulating MAPS and clinical features (including age, subtype, gender and stage) were performed to determine whether the circulating MAPS could be an independent prognostic indicator. Our results indicated that circulating MAPS was an independent indicator that impact OS (MAPS-low vs MAPS-high: HR = 0.065, 95% CI 0.024–0.18, *P* < 0.001), and high pathological stages were associated with poor OS as expected (Stage IV vs Stage I: HR = 6.535, 95% CI 1.632—26.16, *P* = 0.008; Stage III vs Stage I: HR = 5.731, 95% CI 2.061–15.93, *P* < 0.001) (Fig. 3A). Furthermore, we developed nomogram survival models by integrating clinical factors without (Fig. 3B) and with (Fig. 3C) circulating MAPS to predict the 1-, 3- and 5 year OS probability of NSCLC patients. The C-index value of the nomogram models were 0.760 and 0.884, respectively. The time-dependent ROCs were shown in Fig. 3D–E, which indicated that circulating MAPS may improve the prognosis predictive power. In summary, the circulating microbiome profile was significantly associated with the prognosis of patients with NSCLC, and the prediction MAPS model consisting of 14 microbes showed great prognostic value for NSCLC patients.

### Circulating MAPS was closely relevant to clinical features of NSCLC patients

**Fig. 4:**
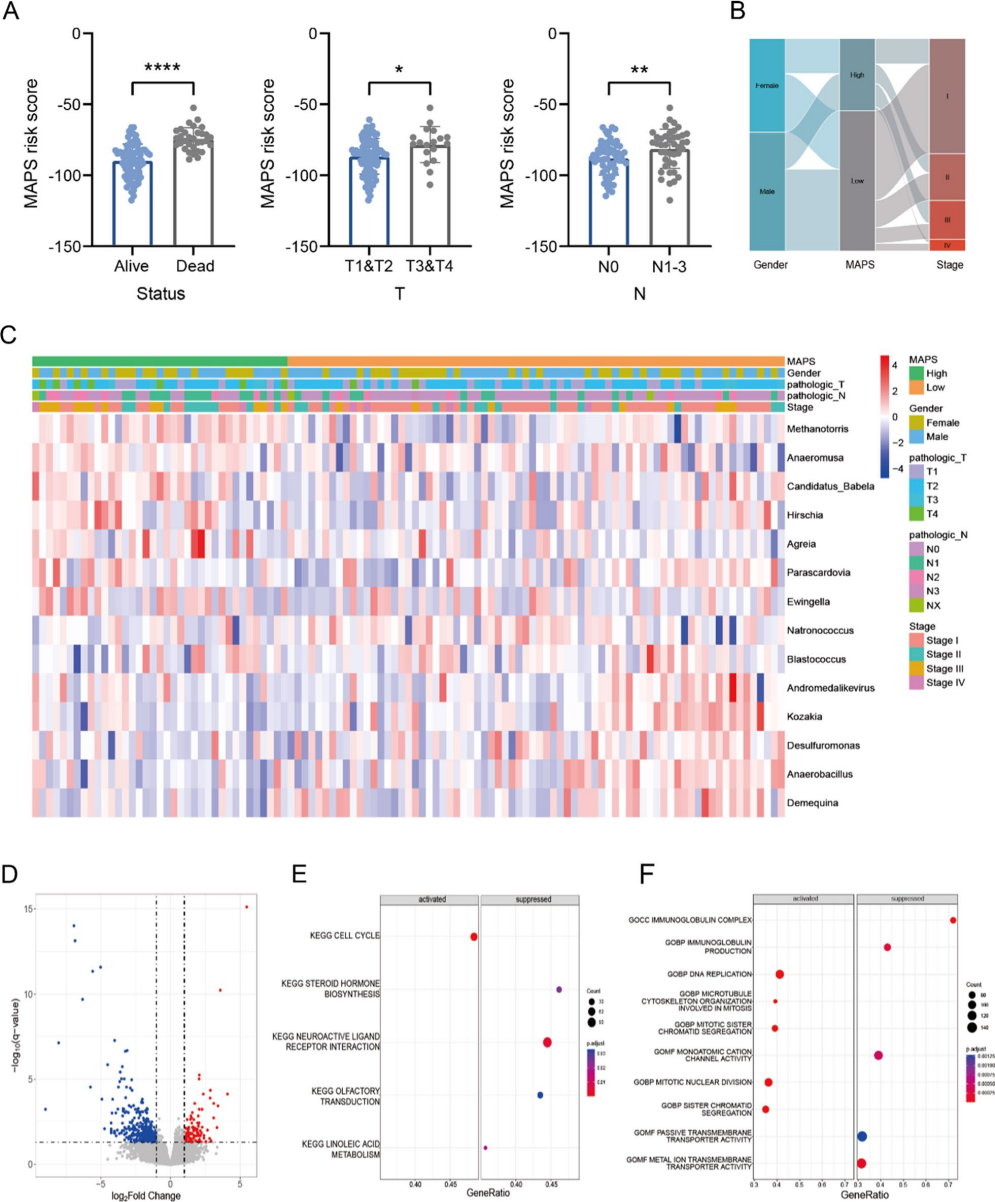
Relationship between circulating MAPS and clinical features of NSCLC patients. **A**, **B** Boxplots and Sankey diagram showed the relationship between circulating MAPS risk scores and clinical features of NSCLC patients. **** *P* < 0.0001; ** *P* < 0.01; * *P* < 0.05. **C** Heatmap of 14 model microbial signatures and various clinical features. **D** Volcano plot showed the differentially expressed genes (DEGs) (*P.adj* < 0.05 and |log_2_ FC|> 1) in tumors between circulating MAPS-high and MAPS-low groups through DESeq2 analysis. **E**, **F** Dot plot showed all items of KEGG enrichment analysis **E** and the top five items in each group of GO enrichment analysis **F** through GSEA

We further explored the relationship between circulating MAPS and clinical features of NSCLC patients. As a result, we found no difference in age (*P* = 0.181) or gender (*P* = 0.487) between circulating MAPS-high and MAPS-low groups. Besides, the circulating MAPS scores of NSCLC patients were significantly correlated with clinical features, including survival outcome (alive or dead), pathologic T stage (T1–T4), and pathologic N stage (N0–N3) (Fig. 4A–C). Based on the matching tumor RNA-seq data of NSCLC patients, we identified 405 differentially expressed genes (DEGs) (*P.adj* < 0.05 and |log_2_ FC|> 1) between circulating MAPS-high and MAPS-low groups, which can well distinguish the two groups (Fig. 4D). As expected, typical proliferative gene sets, such as cell cycle, DNA replication and mitotic nuclear division pathways, were enriched in circulating MAPS-high group through GSEA. Conversely, immune-related gene sets, including immunoglobulin complex and immunoglobulin production pathways, as well as metabolic pathways, such as linoleic acid metabolism and steroid hormone biosynthesis pathways, were enriched in circulating MAPS-low group (Fig. 4E, F). These results suggested that circulating MAPS was closely relevant to clinical features of NSCLC patients and may influence prognosis by regulating proliferation and affecting immune and metabolic functions.

### Joint analysis revealed potential immune-microbe interactions

**Fig. 5:**
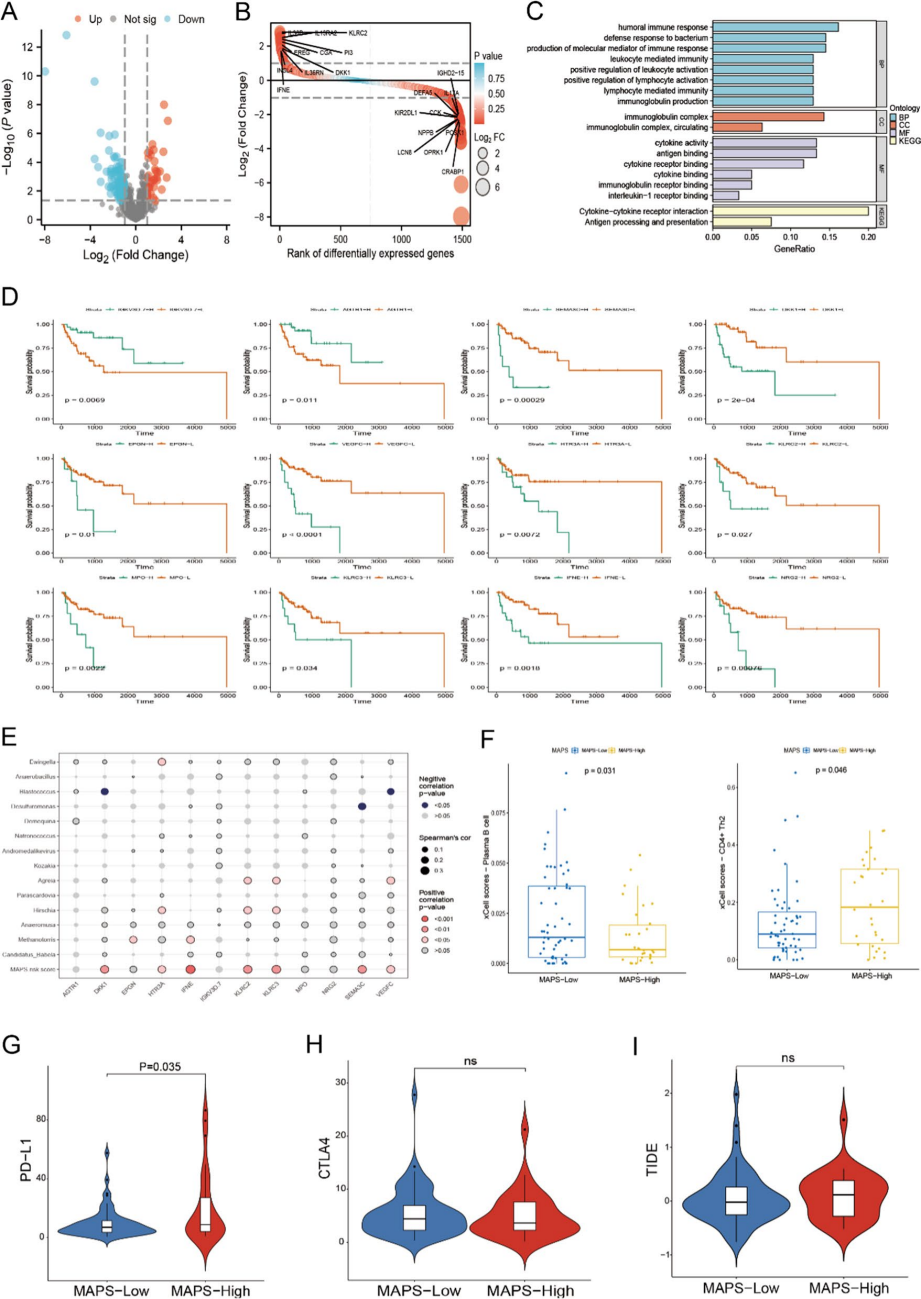
Decipherment of the tumor immune microenvironment based on circulating MAPS. **A**, **B** Volcano plot **A** and ranking chart **B** of the immune-related DEGs in tumors between circulating MAPS-high and MAPS-low groups. **C** GO and KEGG enrichment analysis based on the immune-related DEGs. **D** Kaplan–Meier OS curves of 12 immune-related DEGs. The *P* value was calculated by log-rank test between DEG-high and DEG-low groups. **E** Bubble plot of the relationship between 14 model microbes, circulating MAPS score and 12 OS-related immune DEGs. **F** Differences in xCell scores of plasma B cells and CD4^+^ Th2 cells between circulating MAPS-high and MAPS-low groups. **G**–**I** Differences in PD-L1 expression levels **G**, CTLA4 expression levels **H** and the TIDE scores **I** between circulating MAPS-high and MAPS-low groups

We further deciphered the host’s immune-microbe interactions. According to the ImmPort database, we identified 65 immune-related DEGs (*P.adj* < 0.05 and |log_2_ FC|> 1) in tumors between circulating MAPS-high and MAPS-low groups. The volcano plot and the differential ranking chart of the immune-related DEGs were shown in Fig. 5A, B. Furthermore, GO and KEGG enrichment analysis indicated that these immune-related DEGs are involved in humoral immune response, defense response to bacterium, immunoglobulin complex, cytokine activity, antigen processing and presentation pathways and et al. (Fig. 5C). We further identified 12 immune-related DEGs which were significantly associated with OS of NSCLC patients according to the univariate cox regression analysis and Kaplan–Meier survival analysis (Fig. 5D). Among them, *IGKV3D-7* and *AGTR1* were favorable factors, while *DKK1*, *SEMA3C*, *HTR3A*, *VEGFC*, *KLRC2*, *EPGN*, *NRG2*, *MPO*, *KLRC3* and *IFNE* were risk factors (Fig. 5D). The landscape of the correlation between 14 model microbes, circulating MAPS score and 12 OS-related immune DEGs were shown in Fig. 5E. The circulating MAPS risk score and prognosis risk microbes (*Methanotorris*, *Hirschia* and *Agreia*) were positively correlated with the prognosis risk genes, while the prognosis favorable microbes (*Desulfuromonas* and *Blastococcus*) were negatively correlated with the prognosis risk genes (Fig. 5E). In addition, the tumor immune infiltration analysis revealed that tumors with low circulating MAPS were infiltrated with more plasma B cells, while tumors with high circulating MAPS were characterized by more CD4^+^ Th2 cells (Fig. 5F). Finally, our results showed a significant difference between these two groups for immune checkpoint molecule of PD-L1, but not for CTLA4 (Fig. 5G, H). All results above indicated that the circulating MAPS and 14 model microbes were closely related to immune response of NSCLC patients, especially the humoral immune response.

### Circulating MAPS was related to the efficacy of chemotherapy, but may have a limited competency to predict that of immunotherapy

Recent studies have shown the role of microbes in modulating the response to cancer treatment [13, 22]. Many studies have shown that patients with high PD-L1 or CTLA4 expression levels may benefit more from immunotherapy [23, 24]. We observed significant difference in the immune checkpoint molecule of PD-L1 between circulating MAPS-high and MAPS-low groups (Fig. 5G). Thus, we performed the TIDE immunotherapy response assessment to explore the prediction value of circulating MAPS on immunotherapy. However, we found no difference between circulating MAPS-high and MAPS-low groups (Fig. 5I), suggesting that circulating MAPS may have a limited competency to predict the efficacy of immunotherapy.

**Fig. 6:**
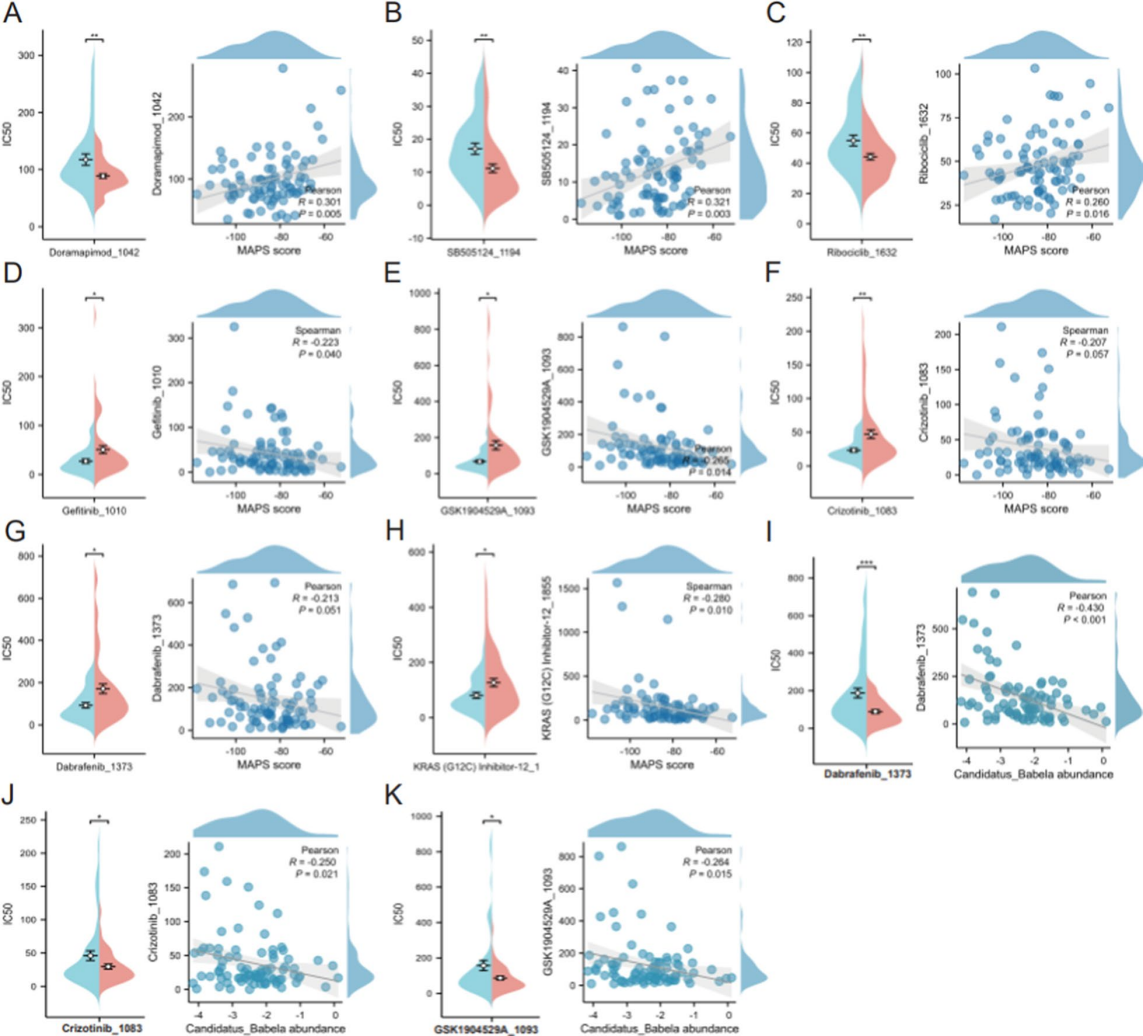
Prediction of chemotherapy drug sensitivity based on circulating microbiome signatures. **A**–**H** Boxplots and correlation plots of the comparison in IC50 values of Doramapimod_1042 **A**, SB505124_1194 **B**, Ribociclib_1632 **C**, Gefitinib_1010 **D**, GSK1904529A_1093 **E**, Crizotinib_1083 **F**, Dabrafenib_1373 **G** and KRAS (G12C) Inhibitor-12_1855 **H** between circulating MAPS-high and MAPS-low groups. The blue box represented circulating MAPS-high group, and the red box represented circulating MAPS-low group. **I**–**K** Boxplots and correlation plots of the comparison in IC50 values of Dabrafenib_1373 **I**, Crizotinib_1083 **J** and GSK1904529A_1093 **K** between circulating *Candidatus_Babela*-high and *Candidatus_Babela*-low groups. The blue box represented circulating *Candidatus_Babela*-low group, and the red box represented circulating *Candidatus_Babela*-high group. *** *P* < 0.001; ** *P* < 0.01; * *P* < 0.05

To further elucidate the relationship between circulating MAPS and chemotherapy drug sensitivity, we predicted each drug’s IC50 value in NSCLC patients by “oncoPredict” package. The results prompted the drugs with significant differences in therapeutic responses between circulating MAPS-high and MAPS-low groups. We found that the IC50 values of three drugs, including Doramapimod_1042, SB505124_1194 and Ribociclib_1632, were significantly higher in circulating MAPS-high group than that in MAPS-low group (Fig. 6A–C), suggesting that NSCLC patients with high circulating MAPS were more resistant to these chemotherapy drugs. Conversely, the IC50 values of five drugs, including GSK1904529A_1093, Gefitinib_1010, Crizotinib_1083, Dabrafenib_1373 and KRAS (G12C) Inhibitor-12_1855 were significantly higher in circulating MAPS-low group than that in MAPS-high group (Fig. 6D–H), suggesting that NSCLC patients with high circulating MAPS may benefit more from these drugs. Besides, we found that circulating *Candidatus_Babela* were also associated with the effect of Dabrafenib_1373, Crizotinib_1083, and GSK1904529A_1093 (Fig. 6I–K), which may contribute to personalized chemotherapy predictions. Interestingly, our results showed that the effect of KRAS (G12C) Inhibitor-12_1855 was closely correlated with age (*R* = 0.239, *P* = 0.028), suggesting that age may also play a non-negligible role in chemotherapy drug sensitivity.

## Discussion

Novel explorations of microbiome signatures provide new ideas for cancer progression. In this study, we identified 14 OS-related circulating microbes to construct a circulating MAPS prediction model and assessed its independent prognostic value in NSCLC patients. Furthermore, we deciphered its potential mechanism on tumor immune microenvironment. Finally, we proposed that circulating MAPS may be a potential biomarker of chemotherapy sensitivity in NSCLC patients. Our study provides a new idea for cancer development and immune-microbe interactions, and may contribute to personalized intervention of NSCLC patients.

Currently, cancer-related biomarkers are mainly identified from genomic or proteomic profiles. In recent years, the previously underestimated role of the microbiome in cancers has been highlighted for its promising potential in cancer development and treatment [13]. Previous studies have confirmed the important role of lung microbiome in lung cancer progression and intervention [10, 11]. However, given the routine inaccessibility of lung tissues, other sources of microbial dysbiosis, such as in the gut, sputum and blood, may be also associated with lung cancer and are attracting more attention [25–28]. For example, Zhang et al. analyzed the gut microbiome composition of lung cancer patients and healthy volunteers, and found higher levels of *Bacteroides*, *Veillonella*, and *Fusobacterium* in lung cancer patients [25]. Hakozaki et al. and Qiu et al. reported that gut microbiome was associated with the response to immunotherapy and chemoradiotherapy in NSCLC patients [27, 28]. However, few studies have explored the relevance between blood microbiome signatures and lung cancer, which is non-invasive and can reflect a general view of the microbiome of host [13]. Only Poore et al. explored the clinical value of cmDNA in the diagnosis of lung cancer by mining TCGA data and training machine learning models [12]. As the first study focusing on the role of circulating microbiome signatures in NSCLC prognosis and tumor immune microenvironment, we uncovered that the circulating MAPS served as an independent prognostic factor of NSCLC and preliminarily revealed the interactions between microbes, tumors and immunity. Among the 14 OS-related microbiome signatures included in MAPS, oral *Parascardovia* was found to be relevant to betel nut chewing, and the latter was an independent predictor of oral premalignant lesions [29]. However, the role of the remaining microbes in the development of cancers has not been reported yet.

Characterizing the circulating microbiome and its underlying mechanism on cancer patients is of great interest. The enormous microbiome of the human body plays a significant role in shaping the host immune characteristics [30–32]. The mutual adaptation process between human microbiome and immune can affect the response of immune system to malignant cells and thus impact the effectiveness of anti-tumor immune response [8]. Previous studies have demonstrated a link between the intratumor microbiome and immune characteristics [9, 26]. On the one hand, the intratumor microbiome may contribute to cancer development by promoting a pro-carcinogenic inflammatory cascade, shaping an immunosuppressive tumor microenvironment or T cell dysfunction [33–35]. On the other hand, the intratumor microbiome can also play an active role in anti-tumor immune response by tumor neoantigen mimicry by microbes, T and NK cell activation, and regulating other immune cells [36–38]. Based on the above studies, we wonder whether the circulating microbiome is relevant to the intratumor immune characteristics, and thus to cancer progression. It’s interesting that we found the intratumor plasma B cells and the humoral immune response related pathways, such as immunoglobulin complex and immunoglobulin production pathways, were enriched in NSCLC patients with low circulating MAPS, who had a longer OS and a better prognostic outcome. Previous studies have found that B cells and plasma cells in NSCLC are associated with better outcomes [39–41]. In recent years, researchers have shown that B cells and plasma cells infiltrated in tumors can play a significant role in shaping anti-tumor immune responses [40, 42]. Plasma cells may produce large amounts of cytokines and tumor-antigen-specific antibodies, leading to antibody-dependent cellular cytotoxicity (ADCC) and complement activation [42, 43]. Besides, B cells can act as antigen-presenting cells (APCs) to present specific antigens to CD4 and CD8 T cells, and thereby maintain and expand long-term antitumor immunity within the tumor microenvironment [44, 45]. It is important to note that the above effective antitumor immune response is premised on the recognition of tumor antigens. The tumor neoantigen mimicry by microbes reported previously may explain one of the mechanisms by which the circulating microbiome participate in the antitumor immune response described above [36, 46]. In addition, it’s well known that Th2 cells contribute to cancer progression and metastasis by secreting IL-4, IL-5, IL-10 and IL-13 [47, 48]. Consistent with this, our study revealed an increased CD4^+^ Th2 cells infiltration in NSCLC tumors with high circulating MAPS. Besides, we found that *DDK1* was associated with poor prognosis of NSCLC patients and positively correlated to circulating MAPS in this study, which have been reported to promote tumor progression and negatively regulate antitumor immunity [49]. In addition, it’s interesting that linoleic acid metabolism and steroid hormone biosynthesis pathways were enriched in circulating MAPS-low group, which may help alleviate the pathological features and improve the anti-tumor ability of patients [50–52]. What’s more, we observed that patients with high circulating MAPS exhibited significant proliferation characteristics. And it may also suggest one of the mechanisms by which circulating microbes impact tumor progression.

Currently, conventional antitumor treatments include chemotherapy, radiotherapy, immunotherapy and et al. However, not all patients benefit from the above therapies, so it’s urgent to explore new prognostic biomarkers for individualized treatment of NSCLC patients. In recent years, studies have reported that microbes play an important role in regulating response to antitumor treatments [22, 53–56]. For example, Fusobacterium nucleatum were found to promote chemotherapy resistance by modulating autophagy [57]. Recently, the gut microbiome has been reported as promising predictive biomarkers of chemotherapy and immunotherapy sensitivity in cancer patients [58–60]. We wondered if the circulating MAPS prognostic score identified in this study could be useful in predicting chemotherapy or immunotherapy sensitivity. Our results suggested that the circulating MAPS may contribute to chemotherapy sensitivity prediction, which may help develop personalized cancer treatment strategies. However, although the expression levels of PD-L1 differed between circulating MAPS-high and MAPS-low groups, the TIDE (Tumor Immune Dysfunction and Exclusion) scores showed no difference between these two groups. The predictive value of circulating microbiome signatures on immunotherapy remains to be further explored.

Although the circulating MAPS model performed well in prognosis prediction of NSCLC patients, there are some limitations deserving further discussion. A major limitation was that there was no additional publicly available dataset to validate our findings. In addition, as a secondary analysis of retrospective data, it’s hard to control the various potential confounding factors among participants. Therefore, it is necessary to design comprehensive prospective multicenter studies to verify the prognostic performance of circulating microbiome signatures. Furthermore, our study preliminarily deciphered the relationship between circulating microbiome signatures and tumor microenvironment and prognosis in NSCLC, while the causal relationship and underlying mechanisms need to be further explored.

## Conclusion

We identified 14 OS-related circulating microbes and successfully construct a circulating MAPS model for prognosis prediction of NSCLC patients. Furthermore, joint analysis revealed potential interactions between circulating microbiome signatures and tumor immune microenvironment, especially the humoral immune response. Finally, drug sensitivity analysis indicated the potential of circulating MAPS as a biomarker of chemotherapy efficacy in NSCLC patients. Our study provides new insights of interactions between microbes, tumors, and immunity, and may further promote the development of precision medicine for NSCLC.

### Supplementary Information

**Additional file1: Tables S1–S3.** Datasets and patients used in this study.

**Additional file 2: Table S4.** Construction of the circulating MAPS model for patients in this study.

## Data Availability

The datasets supporting the conclusions of this article are available in open access (ftp://ftp.microbio.me/pub/cancer_microbiome_analysis/) from Poore’s study and UCSC Xena (http://xena.ucsc.edu/). All the other data of this study are available within the article and its Additional files, or from the corresponding authors upon reasonable request.

## Abbreviations

NSCLC: Non-small cell lung cancer
MAPS: Circulating microbial abundance prognostic scoring
OS: Overall survival
cmDNA: Circulating microbial DNA
KM: Kaplan–Meier
C-index: Concordance index
DEGs: Differentially expressed genes
GSEA: Gene set enrichment analysis
IC50: Drug’s half maximal inhibitory concentration
ADCC: Antibody-dependent cellular cytotoxicity
APCs: Antigen-presenting cells
